# SoyXpress: A database for exploring the soybean transcriptome

**DOI:** 10.1186/1471-2164-9-368

**Published:** 2008-08-01

**Authors:** Kei Chin Christine Cheng, Martina V Strömvik

**Affiliations:** 1 Department of Plant Science, McGill University, 21,111 Lakeshore Rd, Sainte Anne de Bellevue, QC H9X 3V9, Canada

## Abstract

**Background:**

Experiments using whole transcriptome microarrays produce massive amounts of data. To gain a comprehensive understanding of this gene expression data it needs to be integrated with other available information such as gene function and metabolic pathways. Bioinformatics tools are essential to handle, organize and interpret the results. To date, no database provides whole transcriptome analysis capabilities integrated with terms describing biological functions for soybean (*Glycine max *(L) Merr.). To this end we have developed SoyXpress, a relational database with a suite of web interfaces to allow users to easily retrieve data and results of the microarray experiment with cross-referenced annotations of expressed sequence tags (EST) and hyperlinks to external public databases. This environment makes it possible to explore differences in gene expression, if any, between for instance transgenic and non-transgenic soybean cultivars and to interpret the results based on gene functional annotations to determine any changes that could potentially alter biological processes.

**Results:**

SoyXpress is a database designed for exploring the soybean transcriptome. Currently SoyXpress houses 380,095 soybean Expressed Sequence Tags (EST), linked with metabolic pathways, Gene Ontology terms, SwissProt identifiers and Affymetrix gene expression data. Array data is presently available from an experiment profiling global gene expression of three conventional and two genetically engineered soybean cultivars. The microarray data is linked with the sequence data, for maximum knowledge extraction. SoyXpress is implemented in MySQL and uses a Perl CGI interface.

**Conclusion:**

SoyXpress is designed for the purpose of exploring potential transcriptome differences in different plant genotypes, including genetically modified crops. Soybean EST sequences, microarray and pathway data as well as searchable and browsable gene ontology are integrated and presented. SoyXpress is publicly accessible at .

## Background

Microarrays are most often developed from transcript information in the form of EST (Expressed Sequence Tags) sequences [e.g. [1]]. The annotation of those sequences with information on genetics, homology, functions, metabolic regulations and toxicology, are key to unlocking the biological meaning of the microarray results. Since each single microarray hybridization experiment produces massive amounts of data, handling, processing and analyzing the data become challenging tasks and the application of bioinformatics is absolutely essential. It is desirable to store and organize the results in a database, which needs to be extensible and flexible in order to have the capabilities to compare data from different microarray experiments. The Stanford Microarray Database (SMD) [2] is an example of such a resource, developed primarily with Stanford researchers and their collaborators in mind. Many other communities also develop resource databases, building the tools and functions to suit their organism, for example BarleyBase/PlexDB [3] for Barley genomics, MELOGEN [4] for melon genomics and the Tomato Expression Database (TED) [5].

A soybean gene expression database has been published, SGMD (the Soybean Genomics and Microarray Database – please see Availability and requirements for more information) [6]. SGMD stores EST and microarray data to explore the interaction of soybean with the major pest soybean cyst nematode (SCN). The SGMD web interface provides on-the-fly statistics analysis to compare cDNA microarray data, which consists of around 4,000 spots and 20,000 EST sequences from the soybean root libraries [6].

We developed a new database, SoyXpress, with web tools to retrieve and explore the results of Affymetrix microarray experiments, linking also to other soybean genomic information in order to help researchers identify changes in gene expression and determine whether these changes alter biological processes in soybean. We designed SoyXpress for the potential of exploring the entire soybean transcriptome, integrating Affymetrix gene expression data (37,583 soybean probe sets) with 380,095 ESTs from *G. max *and *G. soja*, annotated with metabolic pathways, Gene Ontology terms, with SwissProt identifiers for maximum knowledge extraction. Currently, SoyXpress houses array data from 25 chips, comprising a leaf gene expression profiling experiment including two transgenic and three conventional (non-transgenic) soybean genotypes. SoyXpress is expansible and future gene expression experiments will be integrated.

## Construction and Content

### Schema and implementation: Sequence core tables

**Figure 1:**
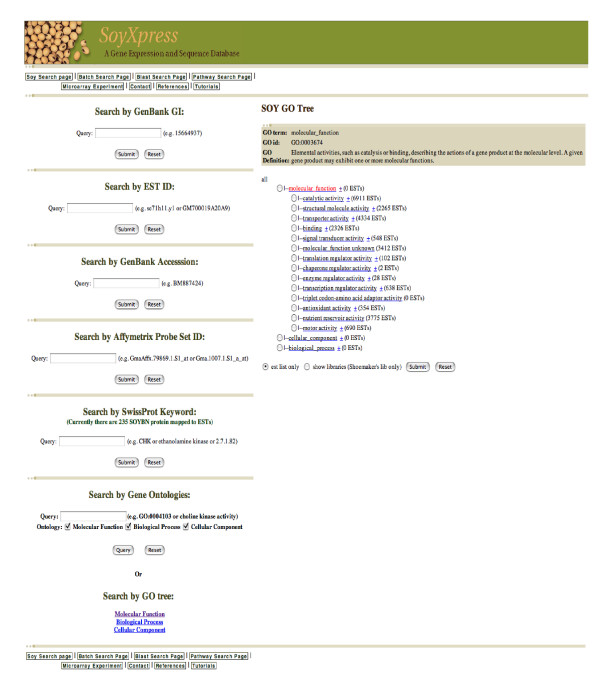
**The main page of the soy database**. Users can submit queries to the database to retrieve all available IDs and annotations for the soybean transcript of interest. Queries can be made using EST ID, GenBank accession number, GenBank GI number, Affymetrix probe Set ID, SwissProt protein ID, name or keyword, GO number or term. A clickable GO tree is available to assist searching for a GO term from the gene ontology hierarchy structure.

**Figure 2:**
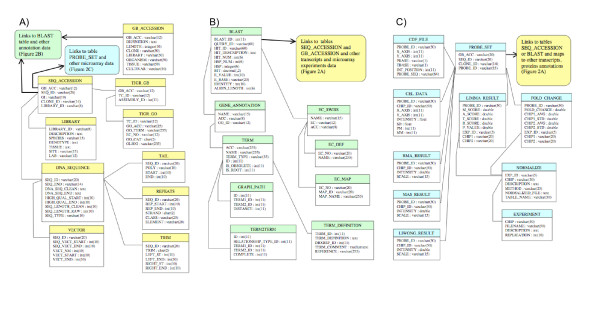
**Database structure**. A) Gene transcript (sequence) information section. Tables for EST sequences (DNA_SEQUENCE, LIBRARY, REPEATS, SEQ_ACCESSION, TAIL, TRIM, VECTOR), a table for mRNA sequences downloaded from GenBank (GB_ACCESSION) and tables for DFCI/TIGR contigs data (TIGR_GB, TIGR_GO) link through the tables GB_ACCESSION and SEQ_ACCESSION to annotation and microarray data. B) Protein and functional annotations section. A table, BLAST, for BLASTX search results, tables for gene ontology terms information (GENE_ANNOTATION, GRAPH_PATH, TERM, TERM2TERM, TERM_DEFINITION) and tables for KEGG pathways with the enzyme commission numbers (EC_DEF, EC_SWISS, EC_MAP) organize the annotation section. The BLAST table links protein annotation data to transcript sequence information and to microarray experiments data. C) Microarray experiment data section. Tables for chip information (CDF_FILE, PROBE_SET), a table for raw data (CEL_DATA), tables for normalized data (LIWONG_RESULT, MAS_RESULT, RMA_RESULT), and tables for analyzed results (EXPERIMENT, FOLD_CHANGE, LIMA_RESULT, NORMALIZE) organize the microarray data section. The PROBE_SET table links the microarray data to transcript sequence information and protein annotations data.

SoyXpress (Figure 1) is implemented in MySQL (version 5.0.18 – please see Availability and requirements for more information). Perl (version 5.8.6) and Perl CGI (please see Availability and requirements for more information) scripts were written for data file parsing, database loading and to create web-interfaces connected to the database using the perl modules DBI and DBD::mysql (please see Availability and requirements for more information). The CGIwithR package [7] was used in order to run R within CGI. The sequence core tables are adapted for MySQL (please see Availability and requirements for more information) from the Oracle-based open source ESTIMA (Expressed Sequence Tag Information Management and Annotation project) database [8]. Figure 2A shows the tables that organize the sequence information. The table DNA_SEQUENCE specifies the sequence ID, length and location (file path) where the sequence is stored in our file system (adapted from the BioData system at the former Center for Computational Genomics and Bioinformatics, University of Minnesota). The ancillary information about the EST sequences such as the locations of the clone vector, polyA-tail, repeat sequence and the trim site are stored in the tables VECTOR, TAIL, REPEATS and TRIM, respectively. The cDNA library information including the library ID, tissue type, and growing conditions are stored in the table LIBRARY. The library information is linked to the sequence information through table SEQ_ACCESSION, which maps the sequence ID, library ID and GenBank accession number.

### Schema and implementation: Sequence annotation tables

Figure 2B shows the annotation section of the database. The SEQ_ACCESSION table links to the BLAST table by using the sequence ID as the query ID for linking to the BLASTX search results. The GenBank accession number from the SEQ_ACCESSION table links to the DFCI (Dana Farber Cancer Institute) Gene Index (formerly TIGR Gene Index [9]) contig information to obtain the corresponding contig ID for the EST sequences (from table TIGR_GB) and the GO terms associated with each contig (from the table TIGR_GO) (Figure 2A). Other information for the additional 8,936 EST sequences downloaded from NCBI websites are stored in the table GB_ACCESSION, which also links to the BLAST table using the GenBank accession number as the query ID. The Gene Ontology databases [10] include the MySQL tables: TERM, TERM_DEFINITION, TERM2TERM, a n d GRAPH_PATH (please see Availability and requirements for more information), which were downloaded and directly reproduced in our database. The BLASTX analysis [11] against SwissProt [12] allowed us to assign protein annotations to 175,910 ESTs (over half of the 318,422 EST sequences). The BLAST table contains the BLASTX search results and links our EST data to their corresponding protein information. Of the 37,637 soybean probe sequences on the Affymetrix GeneChip, we assigned protein annotations to 8,667 sequences. These BLASTX search results are also incorporated into the BLAST table and link to other protein and function annotations. The SwissProt protein names are stored as the hit IDs. Other information about the proteins such as the protein descriptions, hit scores and e-values (negative exponents) are also stored in the BLAST table. The SwissProt protein IDs link to other functional annotations such as gene ontology (GO terms) and KEGG molecular pathways [13] through the GENE_ANNOTATION and EC_SWISS tables. The protein descriptions that describe the enzymes with appropriate EC (enzyme commission) numbers are linked to the KEGG pathways (stored as tables EC_DEF, and EC_MAP) through EC_SWISS table. There are 73,996 EST sequences with assigned EC numbers, around 23% of the EST sequences were enzymes. By linking the transcript sequences data to SwissProt annotations through BLASTX search results in the BLAST table, we can map the transcript sequences to their corresponding functional annotations such as GO terms and KEGG molecular pathways providing a more comprehensive description of the soybean data.

### Schema and implementation: soybean microarray experiment data tables

The section of the database that organizes the microarray data is shown in Figure 2C. Data for the Affymetrix Soybean GeneChip [14], for example the probe IDs, the sequences of the probes, and the locations of the probes on the chip are stored in the table CDF_FILE. The whole transcript sequences representing the genes with the corresponding probe IDs and GenBank accession number are stored in the table PROBE_SEQ. The PROBE_SET table contains the probe IDs, GenBank accession number, and the corresponding sequence and clone IDs to map to our soybean EST data, and hence associates the microarray data with corresponding transcript, protein and functional annotations. Also, the microarray data can directly link to the BLAST table by using probe ID as the query ID to provide biological context for our microarray experiment. The raw data for our microarray experiment are stored in the table CEL_DATA, which contains the information for every chip, such as the chip IDs, probe IDs, and probe intensity. The processed data for our microarray experiment using three normalization methods RMA, MAS, dCHIP are stored in three tables RMA_RESULT, MAS_RESULT and LIWONG_RESULT respectively. All the raw and processed microarray data is linked to the PROBE_SET table by the probe IDs. For the analyzed results, the EXPERIMENT table describes which chips are used for the pair-wise comparison. The NORMALIZE table describes which normalization method are used in each pair-wise comparison. The microarray results for each pair-wise comparison analyzed by the LIMMA package are stored in the LIMMA_RESULT table. It includes the scores and p-value from the statistical test for each probe in all pair-wise comparisons. Also, the fold change and average intensity for each probe in all pair-wise comparisons are stored in the table FOLD_CHANGE. All the analyzed microarray results are linked to the PROBE_SET table and hence integrated with the soybean transcript, protein and functional annotations that can provide insight into biological and functional differences between samples.

### Sequence and microarray data sources

The sequence data annotated and stored in SoyXpress comprises a total of 380,095 public ESTs from *G. max *and *G. soja*. Information on 31,928 tentative consensus (TC) sequences was downloaded from The TIGR (The Institute for Genomic Research) Glycine max Gene Index Project (Release 12.0) [9] (now hosted at the Dana Farber Cancer Institute/Computational Biology and Functional Genomics Laboratory at Harvard University – please see Availability and requirements for more information). The microarray data currently available consists of twenty-five raw data files (CEL files) of an experiment using the Affymetrix Soybean GeneChip [14]. These were pre-processed and analyzed by standard methods as previously described [15]). The data consists of five biological replicates of leaf gene expression measure of three conventional and two genetically engineered soybean lines. The microarray data is accessible at NCBI (Gene Expression Omnibus) GEO under the accession numbers: GSE9374: GSM238030, GSM238031, GSM238032, GSM238033, GSM238034, GSM238036, GSM238038, GSM238039, GSM238041, GSM238043, GSM238047, GSM238048, GSM238049, GSM238050, GSM238051, GSM238052, GSM238053, GSM238054, GSM238055, GSM238056, GSM238057, GSM238058, GSM238059, GSM238060, GSM238061. Microarray chip information (from Affymetrix), raw data and results are stored in SoyXpress, and each probe is linked to the sequence information and meta-data.

### Informatics of data generation and quality control

The EST sequences were annotated by command line BLASTX [11] searches against 168,297 SwissProt protein sequences (please see Availability and requirements for more information) [12] to obtain corresponding protein annotations. The SwissProt protein IDs were used to associate the sequences with GO terms, using the file: "UniProt GO Annotations" (please see Availability and requirements for more information). Recommended enzyme names and EC numbers were obtained from the Enzyme Nomenclature site (please see Availability and requirements for more information) and also extracted from MeSH (Medical Subject Headings, National Library of Medicine – please see Availability and requirements for more information). Enzyme EC numbers to SwissProt ID associations were obtained from the ExPASy Enzyme nomenclature database (version 36, please see Availability and requirements for more information). Metabolic and regulatory pathways were downloaded from KEGG (Kyoto Encyclopedia of Genes and Genomes – please see Availability and requirements for more information [13]). Enzyme identities within each pathway were obtained by extracting EC numbers from each of the pathways (downloadable XML files from the ftp KGML/map folders, version 0.6 Mar 2005). EC numbers, pathway names and map numbers where extracted and integrated into the database.

## Utility and Discussion

**Figure 3:**
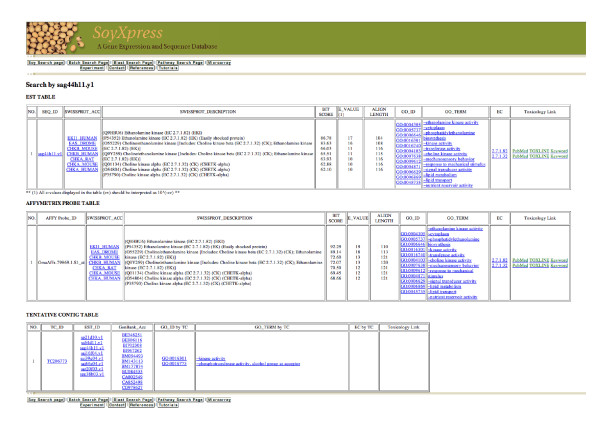
**Database result page, showing information about an EST sequence sag44h11.y1**. Data records are presented in three tables: a) EST table displays the BLASTX result with scores and the negative exponent for the e-value for the EST sequence. b) AFFY table displays the BLASTX result with scores and the negative exponent for the e-value for the Affymetrix probe sequence. c) TC table displays the information for the corresponding DFCI/TIGR contig. The SwissProt protein ID/description, GO number/terms and EC enzyme numbers are displayed with the corresponding EST ID, GenBank accession number or Affymetrix probe ID with hyperlinks to the original public database. If available, links to TOXLINE are also given.

We present SoyXpress, where we have integrated 380,095 soybean EST sequences and Affymetrix microarray data with functional annotations such as metabolic pathways and gene ontology. The SoyXpress user web interface was developed to access the database and display the data in a tabular format. Figure 1 shows the Search Page to retrieve all available IDs and annotations for a soybean transcript or a group of transcripts that share similar protein name or function from our database. SoyXpress can be queried by EST ID, GenBank accession number, Affymetrix probe ID, SwissProt protein ID/name, EC enzyme number or GO term/number. A clickable GO tree that illustrates the hierarchy structure of the ontology is available to select a GO term for searching the associated IDs and annotation for the corresponding soybean sequences from the database. Figure 3 shows the Search Result page for displaying all available IDs and annotations for the (EST) query IDs from the Search Page. After receiving the query ID, the corresponding EST sequence, Affymetrix probe sequence and DFCI/TIGR TC contig will be retrieved from our database. The BLASTX results for the EST and the Affymetrix probe sequences, such as the SwissProt protein IDs and descriptions, BLAST scores and evalues (represented by the negative exponent of the e-value), are displayed in the EST and AFFY tables. The associated GO numbers, GO terms and EC enzyme number are also displayed. The TC table displays the information for the DFCI/TIGR contig, such as TC ID, the IDs and GenBank accession numbers for the EST members of that contig, the associated GO number/term and EC enzyme number. To facilitate detailed database searches for the soybean search results, all these IDs are hyperlinked to the original public databases, such as GenBank (please see Availability and requirements for more information) for the sequence information, the SwissProt protein database (please see Availability and requirements for more information) for the protein information, the Gene Ontology (please see Availability and requirements for more information) for the functional annotations, the KEGG database (please see Availability and requirements for more information) for the biological pathway maps and enzyme information and the DFCI/TIGR Gene Index for the contig sequence and information (please see Availability and requirements for more information). The information on the cDNA libraries are presented on static html pages adapted from the former Soybean Genomics Initiative website (at the former Center for Computational Genomics and Bioinformatics, University of Minnesota).

**Figure 4:**
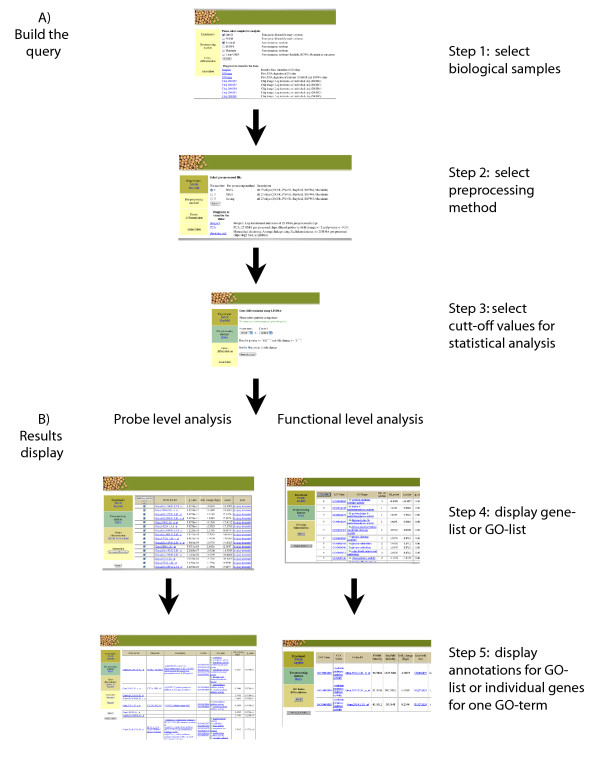
**Flowchart of the microarray web interface to access the database for displaying differentially expressed genes or functional gene classes based on GO terms**. A) The query is built in three steps. B) There are four different views for the query results: by probe or functional level analysis either displayed as a list or as more informative annotated list.

The results for the microarray experiment can be retrieved from the database through a special section of the interface. An overview of the query flow is presented in Figure 4. At the top SOY Microarray Analysis webpage, any of the five soybean cultivar samples can be selected for pair-wise comparison (Step 1). Diagrams to assess the quality of the data, such as boxplots of probe intensities, RNA degradation plots and the individual chip images are presented. After selecting two samples, the web page allows a choice of normalization method for pre-processing the raw data (Step 2). Diagrams such as boxplot, PCA analysis and hierarchical clustering are available to visualize the preprocessed data. After selecting the pre-processing method, the webpage allows selecting the cut-off p-value and fold change for differentially expressed genes from the results of the statistical analysis (Step 3). The list of differentially expressed genes from the pairwise comparison is displayed, ordered by probe IDs (Step 4). Statistical scores such as t-score, p-value and fold change are also displayed. A hyperlink is provided to display a plot of the intensities of an individual probe against five soybean cultivars. Check boxes are also available to submit a list of probe IDs to the Soybean Search Page to retrieve all the available IDs and annotations for those probes. It links to the annotation view by clicking on the Annotated Probe List button on the left panel. The annotation view (Step 5) displays the associated SwissProt protein ID/description and the GO number/term with the fold change and p-value for the list of differentially expressed genes. All these IDs are hyperlinked to the original public databases to facilitate detailed database searches. To retrieve results from the gene class analysis based on GO term annotations, similar query pages are developed (Figure 4). The list of GO terms (which represents changes of the gene class) is displayed in the result pages with the statistical scores and the number of the genes involved in each gene class. The intensities of the individual genes of each identified GO term can be displayed with log2 fold change, which can allow users to identify whether the genes were regulated in a similar pattern. A special page for BLAST analysis against the sequences from the Affymetrix probe set is also available in order for users to see whether their gene sequence of interest is present in the probe set.

SoyXpress was developed with two main types of users in mind: researchers and regulators with scientific background. Researchers can explore the annotated sequences in a way that relates to their metabolic pathway or process under study. For instance, a researcher interested in the flavonoid pathway could instantly retrieve all ESTs known from soybean that match the enzymes in this pathway. Regulators are asking for tools to help in assessment of novel crops, be they transgenic or obtained by conventional breeding. With SoyXpress, we have provided such a tool, where differences in global gene expression can be compared between any cultivars or groups of cultivars and where the gene expression is linked to metabolic pathways and to literature resources such as PubMed and TOXLINE. This can help regulators decide on whether a novel cultivar is, at the gene expression level, substantially equivalent to conventional cultivars that are generally recognized as safe (GRAS).

There is no other web-based database that makes the soybean transcriptome available. The SGMD is limited to 4000 genes expressed in root and has the aim to explore the Soybean Cyst Nematode and soybean interactions [6]; there are only GenBank IDs and BLASTX reports to show the homology of genes and proteins, and no annotations are provided to give information of the biological function and metabolic pathways. The draft of the soybean genome sequence was announced in January 2008 (please see Availability and requirements for more information), and it is envisioned that SoyXpress can be a helpful tool for the soybean genome annotation phase. Predicted genes can be compared with the information in SoyXpress and more reliable annotation can follow.

Planned future developments of SoyXpress include addition of promoter motif information, UTR features and links to the soybean genome sequence. It is also our hope that other groups will want to house their Affymetrix soybean data in SoyXpress, and an online submission protocol is planned.

## Conclusion

Our scope was to develop a database with a suite of web interfaces to allow users to easily retrieve data and results of microarray experiments with cross-referenced annotations of the expressed sequence tags (EST) and hyperlinks to external public databases. The SoyXpress environment is the most comprehensive bioinformatics tool to date for soybean gene expression analysis and it makes it possible to explore differences in gene expression and to interpret the results based on gene functional annotations to determine any changes that could alter biological processes.

## Availability and requirements

Project name: SoyXpress: a database for the soybean transcriptome

Project home page: .

Operating system: Platform independent

Programming language: Perl

Other requirements: None

Licence: None required

Any restrictions to use by non-academics: None

SGMD (the Soybean Genomics and Microarray Database): 

MySQL (version 5.0.18): 

Perl (version 5.8.6): 

Perl CGI: 

DBI and DBD::mysql: 

MySQL: 

Gene Ontology databases: 

Glycine max Gene Index Project: 

SwissProt: 

UniProt GO Annotations: 

Enzyme Nomenclature site: 

MeSH (Medical Subject Headings, National Library of Medicine): 

ExPASy Enzyme nomenclature database (version 36): 

KEGG (Kyoto Encyclopedia of Genes and Genomes): 

GenBank: 

SwissProt protein database: 

Gene Ontology: 

KEGG database: 

DFCI/TIGR Gene Index: 

Draft of the soybean genome sequence: 

## Authors' contributions

KCCC designed the database, performed the data analyses and web implementation. MVS conceived and designed the overall project, and assisted in the database design. Both authors have participated in writing the manuscript and have read and approved the final submitted version.
